# Workplace Violence, Self-Perceived Resilience and Associations with Turnover Intention Among Emergency Department Nurses: A Cross-Sectional Study

**DOI:** 10.3390/healthcare13202562

**Published:** 2025-10-11

**Authors:** Anna T. El Riz, Maria Dimitriadou, Maria Karanikola

**Affiliations:** Nursing Department, School of Health Sciences, Cyprus University of Technology, 3036 Limassol, Cyprus; annaelriz141200@hotmail.com (A.T.E.R.); maria.dimitriadou@cut.ac.cy (M.D.)

**Keywords:** workplace violence, nurses, resilience, emergency care, emergency department, assault, Cyprus

## Abstract

**Background/Objectives**: Workplace violence remains an important vocational psycho-social risk for nurses employed in the emergency department (ED). We investigated the characteristics of workplace violence against ED nurses, and associations with self-assessed resilience, socio-demographic and vocational parameters, including turnover intention. **Methods**: ED nurses employed in all public hospitals in the Republic of Cyprus (RC) participated. After obtaining informed consent, data were collected using census sampling (January–June 2024) via the translated 2016 Italian National Survey on Violence towards Emergency Nurses Questionnaire (QuINVIP16) for investigating workplace violence characteristics, and the Connor-Davidson Resilience Scale (CD-RISC-25) for assessing self-perceived resilience. **Results**: A total of 132 nurses (53.0% response rate) participated. Verbal violence was reported by 70.5% to 92.4% of participants. Long waiting times, overcrowded EDs, and perception of inadequate attention from healthcare professionals were reported as the primary triggers for violence towards participants by patients/visitors. One-third of participants reported that violence-reporting systems were unclear, while 1 out of 4 reported inadequate safety measures against violence. Participants with higher scores of self-perceived resilience were less likely to report turnover intention due to workplace violence (*p* < 0.001), while those with lower self-perceived resilience reported a significant decrease in work motivation (*p* = 0.005). Those who experienced decreased work motivation after exposure to a violent episode were more likely to consider a) leaving the profession [OR (95%CI): 79.1(17.7–353.2); *p* < 0.01], and b) moving to a different work setting [OR (95%CI): 17.0(3.8–76.2); *p* < 0.01], and actually applying to be transferred to a different work setting [OR (95%CI): 19.6(4.2–91.5); *p* < 0.01]. Moreover, those who had not attended communication skills training were 4 times more likely to consider leaving the profession following exposure to violence [OR (95%CI): 4.2(1.1–16.2); *p* = 0.04]. **Conclusions**: This study is among the few to link workplace violence with both resilience and actual turnover behaviors among emergency nurses, in general and particularly in the post-pandemic era. By showing how personal resilience in the face of violence is shaped by organizational support, such as reporting systems and training, the present findings move beyond individuals-level explanations, and highlight workplace violence as a systematic administrative challenge. This insight represents an important advance in current knowledge, and calls for multifaceted interventions that strengthen both personal and institutional capacity to address violence.

## 1. Introduction

Workplace violence against healthcare professionals is alarmingly prevalent worldwide, thus broadly recognized as a serious public health concern [1,2]. According to the most widely accepted definition endorsed by the International Labor Organization (ILO), the International Council of Nurses (ICN), the World Health Organization (WHO), and the Public Services International (PSI), workplace violence refers to work-related incidents of abuse, which at the same time encompasses an explicit or implicit challenge to staff’s safety, well-being and health [3]. Workplace violence includes physical assaults (e.g., hitting, biting, pushing), and psychological forms such as verbal abuse, harassment, and threats [4].

According to the WHO, nearly 60% of healthcare professionals experienced some form of violence in 2022 within their work environment [5]. Yet, these incidents are underreported, since less than 40–45% of the staff formally reports such incidents, depending on setting [6,7,8]. Compared to other healthcare workers, those employed in emergency department (ED) are more vulnerable to workplace violence by patients and their relatives, due to the volatile mix of emotional stress, long waits, intoxicated or unstable patients, communication breakdowns, and limited security [9,10,11,12,13]. A systematic review and meta-analysis estimated verbal violence prevalence against ED staff at 77% and for physical violence at 18% [9].

Indeed, systematic reviews and large-scale studies consistently highlight that ED staff experience substantially higher rates of workplace violence compared to general wards or outpatient setting employees, often reporting prevalence rates more than double than those seen in non-ED environments [9,12,13,14]. Among them, nurses are at even greater risk for exposure to workplace violence compared to other ED healthcare employees [15,16,17] due to their frontline role, direct and frequent interactions with patients and their families, and the emotional intensity of care situations [16,18]. For instance, a 2024 survey by the Emergency Nurses Association (ENA) reported that over half of ED nurses had experienced physical or verbal assault or threats within the preceding 30 days highlighting the severity of the issue [19].

Compared to private, public sector healthcare facilities are more susceptible to workplace violence incidents [20,21,22,23], mainly due to larger patient volumes and insufficient supporting resources [10,24]. Additional factors include staff shortages, overcrowding and long waiting times, high workload, high patient turnover, unpredictability of medical emergencies, and inappropriate attitudes and/or ineffective care approaches by employees [25,26,27,28].

This widespread phenomenon raises concerns due to its harmful physical, mental, psychological and organizational consequences. Affected healthcare workers often report diminished self-esteem, increased stress, burnout, poor work performance [29], absenteeism, and turnover intention [25,30]. These outcomes ultimately compromise employees’ health, the quality and safety of patient care [31,32,33,34,35,36], and lead to significant financial burden for healthcare systems [37].

Yet, data on workplace violence against nurses employed in public EDs in the post-pandemic period are limited [38]. In light of the increased physical and verbal assaults, and psychological violence against ED healthcare professionals since the onset of the COVID-19 pandemic [39], further research of the issue in the post-pandemic context would be valuable. Moreover, considering that the occurrence of violence is related to culture [40], so far, studies have come from countries in North America [41,42,43], central Europe [6,44] and Asia [15,45,46], and seem to be limited in Eastern Mediterranean and South-Eastern European countries [12,47]. Studies from multiple cultural contexts are expected to enrich current knowledge on the topic and effectively support the creation of sustainable intervention strategies. Similarly, studies associating the impact of workplace violence with personal characteristics, such as resilience, and professional attitudes, such as intention to quit the job, may inform targeted supportive interventions to high-risk populations.

Indeed, although most workplace violence research emphasizes preventive measures, there is also increasing attention on the factors which may mitigate its adverse effects [48]. Among these, personal resilience [49] and supportive human resource management practices stand out [50,51]. Although resilience is broadly understood as one’s capacity to adapt and recover in the face of adversity [52], it mainly refers to individuals’ ability to evolve and expand their competencies for sustainability after exposure to severe stress and trauma [53]. It is considered a multidimensional personality trait, encompassing adaptability, coping skills, optimism, and self-efficacy [54]. In nursing, resilience is described as a dynamic process that helps clinicians to effectively cope with the adverse effects of prolonged exposure to workplace stressors and recover, also expanding their personal and professional well-being and subsequently providing consistent quality care [55,56]. Higher resilience has been linked to lower burnout, stronger engagement with the profession, greater empathy and self-confidence, and improved clinical performance [57,58,59,60,61,62], and ultimately improved patient care [63]. Resilience has also been shown to buffer emotional exhaustion, enhance job satisfaction, and augment patient safety in frontline healthcare professionals [64].

While personal resilience highlights the importance of individual-level protective resources, outcomes of workplace violence, beyond the severity of exposure, are also shaped by the broader organizational context. According to stress and coping theoretical frameworks, at the individual level personal resilience functions as a psychological resource that enables nurses to cope with workplace stressors, minimize the depletion of emotional energy, and preserve recovery resources [65]. Beyond the individual level, resources available in the work environment also influence the impact of workplace violence, as described by the Conservation of Resources Theory [66]. This theory describes that organizational moderators, such as managerial support, effective reporting systems, and protective policies, serve as structural resources that can either amplify or mitigate the negative effects of workplace violence [67].

Differentiating these individual and structural mechanisms provides conceptual clarity and helps to explain how personal and organizational factors interact in influencing nurses’ well-being, performance, and retention. Yet, despite increasing interest on the factors that may moderate the adverse effects of workplace violence, data specifically addressing the link between workplace violence and personal resilience in ED nurses remains limited [68]. According to a study conducted in 670 ED nurses in China, the importance of resilience in mitigating the adverse effects of workplace violence on them was demonstrated [69]. However, most studies on this association focus on general nursing populations [70,71] or mental health settings [49], yet these results may not be directly applicable to the emergency services context, due to possible different risk factors for workplace violence in this particular setting [49]. In addition, research on this topic from South-Eastern Europe and the Eastern Mediterranean is scarce [12,47].

Addressing the research gap on the association between workplace violence and resilience in ED nurses, the aim of the present study was to investigate the prevalence and forms of workplace violence against emergency care nurses employed in public hospitals in the RC, and to explore the relationship between workplace violence characteristics and self-perceived resilience. Findings from this study are expected to contribute to both the local and international discourse on the post-pandemic workplace violence profile, and the link with factors associated with ED nurses’ well-being, such as resilience. Specifically, relevant data may be applicable both locally and across the wider European healthcare landscape in improving the quality and safety of ED nurses’ work environment, factors strongly associated with professional retention and sustained engagement [28,72]. Research on ED-related workplace violence and associations with adverse professional attitudes is expected to guide evidence-based policy reforms, institutional strategies, and preventive interventions. Such efforts are instrumental in maintaining a committed nursing workforce, thus ensuring high-quality and safe care, and equally important in promoting nurses’ well-being and optimal working conditions.

### Background

Given the cultural context of emergency care delivery in the RC, and particularly the frequent presence of patients’ relatives during treatment [73,74], as well as the absence of a targeted legislative framework addressing workplace violence, the need for localized empirical evidence becomes even more pressing. Specifically, there is no specific legislative framework dedicated to addressing workplace violence in the RC. Current protections are governed under Law 58(I)/2004 “On Equal Treatment in Employment and Work”, which prohibits discrimination based on religion, belief, age, sexual orientation, gender, or nationality. This law also recognizes harassment as an act which violates a person’s dignity and creates a hostile work environment [75]. These particular contextual dynamics may not only shape the frequency and nature of violent incidents but also influence the coping mechanisms employed by ED nurses.

Moreover, recent studies have highlighted how administrative and organizational factors significantly impact nurses’ resilience [76,77]. Specifically, lack of perceived organizational support has been found to disempower nurses’ ability to recover from workplace violence and reduce their commitment to their professional role [77]. Indeed, structural equation modeling by Tao and colleagues revealed that perceived organizational support positively influenced resilience, which in turn mediated the relationship between exposure to violence and professional identity [77]. Similarly, Huang et al. [76] found that higher self-perceived resilience scores were associated with stronger perceived organizational backing and less vulnerability to the psychological toll of workplace violence. These findings underscore the crucial role that institutional responsiveness and supportive leadership play in buffering the adverse effects of violence and sustaining resilience among emergency nurses [76].

Overall, although workplace violence against healthcare workers has been widely documented, most studies have either focused on its prevalence [9,41] or on individual coping strategies and its psychosocial and mental implications [71], in isolation. Far less is known about how resilience interacts with organizational factors—such as reporting systems, training, and institutional support—and how these dynamics influence work-related motivation and both turnover intentions and actual turnover behaviors [69,71,76,77]. Specifically, the present study aimed to (i) examine the prevalence and characteristics of workplace violence among emergency nurses in the RC, including reporting practices, organizational support, and training; and (ii) explore the associations between workplace violence, self-perceived resilience, and both turnover intentions and actual turnover behaviors, by taking into account organizational factors, such as reporting systems, support and continuing education in preventing violence. By addressing these objectives, this study contributes evidence to guide multifaceted interventions that strengthen resilience, improve organizational responses, and ultimately safeguard the emergency nursing workforce. Most importantly, by investigating these phenomena within the context of the RC, this study aims to fill a critical gap in the post-pandemic literature and offer insights that are both locally relevant and globally informative. Previous studies based on post-pandemic data come from Asia [45,77] and Spain [71]. Ultimately, the findings are anticipated to inform culturally sensitive interventions and policy recommendations, contributing to a safer and more supportive working environment for ED nurses in the RC and similar Mediterranean healthcare systems.

## 2. Materials and Methods

### 2.1. Aim

The aim of the present study was to investigate the characteristics of workplace violence against ED nurses, and associations with self-assessed resilience, socio-demographic and vocational parameters. The following objectives were set: assessment of (i) the prevalence, characteristics and context of workplace violence, including organizational/administrative-level security strategies for managing violent incidents and reporting; (ii) the degree of self-perceived resilience; and (iii) the associations among workplace violence, self-perceived resilience, and both turnover intentions and actual turnover behaviors.

### 2.2. Research Design

The present study followed a quantitative, descriptive correlational design, structured in accordance with Stroke checklist [78].

### 2.3. Sample

The target population included all nurses working in public EDs across RC. A census sampling method was employed to ensure comprehensive coverage and achieve the required sample size, estimated at approximately 150 participants. This estimate was based on a power analysis using Cohen’s statistical method and the G*Power tool, version 3.1. [79], considering the following parameters:

(a) The expected correlation strength r ≈ 0.4, based on previous related studies [9,80];
(b) The size of the desired statistical significance at α = 0.05;
(c) A power index of 0.8 (80%);
(d) The expected comparisons between approximately 20 variables.

According to data provided by the nursing administration of each hospital, the total number of eligible nurses was approximately 249. Considering an anticipated response rate of around 60%, as reported in previous studies [73], all nurses employed in the EDs of all seven public hospitals under the State Health Services Organization (SHSO) (Nicosia, Limassol, Troodos, Polis Chrysochous, Larnaca, Paphos, and Famagusta) were invited to participate.

### 2.4. Data Collection

Data collection was conducted from January to June 2024. Inclusion criteria were: (a) employment in ED for more than 12 months, a criterion relevant to the assessment tool for exposure to workplace violence, i.e., “In the last 12 months, have you experienced violence in your workplace?”; (b) comprehension of the Greek language, since the data collection tool was in Greek; (c) written informed consent. There were no additional exclusion criteria.

Recruitment was facilitated through posters hand-delivered to the heads of the EDs, each containing a QR code linking to the study questionnaire. These posters were displayed in accessible areas (e.g., noticeboards, staff kitchens, supervisors’ offices), and the questionnaire could be completed at any time and place chosen by the participants. The QR code was easily scanned through a mobile phone, and the survey questionnaire through google form application. Although the QR code mode of data collection initially specified that participants should own a mobile phone, this requirement was intended only to facilitate distribution of the electronic survey link. In practice, however, both electronic and paper-based versions of the questionnaire were provided, and no participant was excluded for lack of mobile phone access. Specifically, due to limited participation and technical issues related to QR code scanning, the research team decided to distribute in parallel a paper version of the questionnaire. The principal investigator provided verbal information during poster distribution regarding the study’s purpose, confidentiality, anonymity, voluntary participation, and the right to withdraw at any time without consequence. This information was also included in the consent form, which accompanied each questionnaire. Participants were explicitly instructed not to complete both formats, i.e., printed and electronic, to prevent duplicate responses. This warning was also included in the printed questionnaires, and it was similarly orally addressed during questionnaire distribution and during reminders for participation in the study. Reminders were issued by department heads and through follow-up visits by the principal investigator.

Each electronic submission generated a notification to the main researcher’s email. Upon accessing the electronic questionnaire, the informed consent page of the study was displayed. Subsequently, the participant had to either consent or withdraw. In case the participants to be consented, they were directed to the data collection questionnaire. Otherwise, the google form page was eliminated.

Similarly, each printed questionnaire was accompanied by a consent form which explained the study’s aim, the voluntary nature of participation and guaranteed anonymity and confidentiality. Participants had to personally fill in the date on the consent form to confirm that they had read it and provided consensus to participate in the study. Participants’ names were not recorded at any part of the questionnaire. Completed questionnaires were returned in sealed, non-transparent envelopes, and were collected in person by the main researcher (AE).

### 2.5. Instruments Description

Each questionnaire was self-reported and it comprised 3 parts: (a) demographic (gender, age), vocational (total work experience as a nurse, total work experience in the ED, ranking, work shifts, number of patients served daily in the ED, implementation of patient triage protocol, province/working hospital, availability of architectural, regulatory security measures in the ED, available ED resources for violence response, workplace violence reporting procedures) and educational (highest level of nursing education, education in communication skills, education on workplace violence management) characteristics of participants, (b) the translated in Greek and validated in the context of the RC *“2016 Italian National Survey on Violence towards Emergency Nurses Questionnaire (QuIN16VIP)”* for the measurement of the characteristics of workplace violence [81], and (c) the Greek version of the *“Connor-Davidson Resilience Scale (CD-RISC-25)”* [82] for the measurement of self-perceived degree of resilience.

The QuIN16VIP tool was developed by Ramacciati et al. [81]. It is a self-administered questionnaire developed specifically to investigate workplace violence among emergency nurses. It consists of 39 items and 10 sub-items using closed-ended or multiple-choice questions in the form of a checklist, as well as one open-ended question calling the participants to briefly describe their perception of workplace violence. The following dimensions of workplace violence are explored: exposure to violence, perpetrator characteristics, direct effects of violent acts, violence reporting systems, specific training on violence management, and consequences of violent acts. The mean completion time of the tool is 14 min. A test–retest reliability score measured by the Cohen-Fleiss K coefficient was 0.71 [81].

The Greek version of the CD-RISC-25 consists of 25 items, which assess individuals’ perceptions on their ability to cope with and/or recover from challenging experiences over the past month [82,83]. Each item is rated on a Likert scale from 0 (Not at all true) to 4 (Almost always true), with a cumulative score based on the sum of all items, ranging from 0 to 100. The higher the score the higher degree of self-perceived resilience. The CD-RISC-25 tool has been shown to have high internal consistency reliability (Cronbach’s alpha = 0.925) as well as test–retest reliability (intra-class correlation coefficient = 0.925). Adequate construct validity based on exploratory factor analysis and convergent validity, through correlation with other tools, have also been confirmed for the Greek version of the tool [83].

### 2.6. Cultural Adaptation

Cultural adaptation was conducted solely for the QuIN16VIP tool, as the CD-RISC-25 had already been translated and validated in the Greek language [83]. The QuIN16VIP was initially translated from English into Greek and then back translated into English by a team of four experts. This team included the principal researcher (AE), who was a postgraduate student in Advanced Nursing Practice, and three bilingual academics with backgrounds in social, health, or basic sciences, all of whom had prior experience in instrument translation and adaptation. The expert panel compared the translated version with the original questionnaire and held discussions to resolve any discrepancies related to wording, phrasing, and conceptual meaning, aiming to reach consensus on the final Greek version. A second independent back-translation was conducted to ensure consistency and equivalence. Subsequently, one expert from the translation team conducted an independent review of both the Greek and original English versions to confirm linguistic accuracy and cultural appropriateness. Based on this feedback, and following a final review by the research team, the translated version was deemed ready for use in the next phase of the study.

During the pilot test of the Greek version of the questionnaire, interviews with 6 registered nurses were carried out by a member of the expert group, aiming to assess questionnaire’s readability and comprehension of individual items. During the interviews, participants were asked to provide in-depth insights on the content of each item of the questionnaire to ensure that all questions and response options were clear and easy to understand (face validity), and well-adapted to the local language, cultural context, and healthcare system. Detected errors/misunderstandings identified were minimal, making any changes unnecessary. These six nurses were excluded from the study’s sample.

### 2.7. Ethical Issues

Permission to conduct the study was obtained from the Cyprus National Bioethics Committee (CNBC) (EEBK.EΠ 2023.01.251) and the Research and Innovation Office of the SHSO in order to conduct the study in relevant hospitals. The Scientific/Medical Directors of the hospitals, the Executive Directors of the Divisions and the Heads of Departments were informed about the purpose of the study and received the research protocol. Authorization to access the public EDs was also obtained from the head of each setting. The heads of the EDs and employed nurses were informed verbally by the principal researcher (AE) during the distribution of the posters about the purpose of the research, anonymity, safeguarding the confidentiality of information, and the right to withdraw from the study at any time during the completion of the questionnaire, with absolutely no repercussions being confirmed.

Electronic informed consent was obtained, where participants were informed of their rights on the home page of the link, and after giving their consent, they could proceed with completing the questionnaire. The consent form noted the purpose of the research, the voluntary nature of participation, the preservation of anonymity, the confidentiality of the information, since only the research team would have access to it, and the right to withdraw from the study at any stage, and that no personal data will be requested at all, except for some basic demographic information, specifically years of professional experience, age, gender, highest level of education, etc. Participants had the right to discontinue their participation at any time, without any consequences. After the questionnaires were electronically sent or received by the principal researcher (AE), it was not possible to cancel participation due to anonymization. Participants were asked to complete the questionnaire only once. This information was included in the consent form. The contact details of the principal researcher (AE) in case participants had any questions about the questionnaire or the procedure were also written on the consent form. The principal researcher (AE) received a notification to her personal email address for each completion of an electronic questionnaire. The data were sent directly to the personal account of the principal researcher (AE). Furthermore, the computerization of the data was carried out exclusively by the principal researcher (AE) with complete confidentiality. Only the principal investigator (AE) and the study research team had access to the data.

Permission to use the questionnaires was obtained by each developer.

### 2.8. Statistical Analysis

All statistical analyses were performed using IBM SPSS Statistics (version 25, IBM Corp., Armonk, NY, USA). Descriptive statistics, such as frequencies, % percentages, mean value and standard deviation [M(SD)] scores, were calculated to describe the demographic and study-specific variables. The distribution of variables was checked for normality and parametric tests were applied accordingly. To examine differences in resilience scores across categorical variables, independent samples *t*-tests were used for comparisons between two groups, while one-way analysis of variance (ANOVA) was conducted for variables with more than two groups. Post hoc tests were applied appropriately to identify specific group differences. In addition, a series of binary logistic regression models were conducted to investigate the relationship between exposure to workplace violence, perceived organizational support, participation in communication programs, and self-perceived resilience, using the Forward method: Likelihood Ratio. The dependent (outcome) variables included (a) consider leaving the nursing profession, (b) consider moving to a different work setting, and (c) actually applying to be transferred to a different work setting. The Forward Likelihood Ratio stepwise method was chosen to identify the most parsimonious set of predictors associated with the outcome variables, given the exploratory nature of this study and the relatively large number of candidate variables. While stepwise procedures are not without limitations, they are commonly used in exploratory contexts to reduce model complexity and highlight variables of greatest predictive value.

For the CD-RISC-25 tool, scores were interpreted with a median and quartiles. The quartiles describe four equal groups taken from the observed distribution of scores, with the first quartile (Q1) describing the range of scores for the lowest group (lowest 25% of the population), which is the least resilient, the second (Q2) and third (Q3) are the intermediate scores and the fourth (Q4) describes the highest or most resilient group, in the top 25% and above 75% of the population. The significance level was set at α = 0.05, and all tests were two-tailed. Regarding missing values for the CD-RISC-25 tool, missing responses were scored using the average of the other items. For the scale to be considered valid, at least 75% (19 items) had to be completed. Anything less than this was considered an invalid assessment.

Since the QuINVIP16 instrument is structured as a checklist, in which respondents indicate the presence or absence of specific types/characteristics of violent incidents, the validity of the instrument has been established through prior applications in similar healthcare contexts and was further supported in the present study by a systematic cultural adaptation process, as described above.

An open-ended question was included at the end of the questionnaire to enable participants to elaborate on their earlier responses or to express additional thoughts and personal reflections on workplace violence. The responses were analyzed using descriptive content analysis. Initially, all textual data were reviewed to identify recurring concepts and meaningful segments. Responses were then grouped into thematic categories based on the content and context of participants’ statements. The relative frequency of comments within each category was calculated and expressed as percentages to identify the most prevalent themes.

## 3. Results

### 3.1. Demographic, Vocational and Educational Characteristics of Participants

A total of 132 out of 249 nurses of the target population participated in the study, yielding a response rate of 53.0%. The mean age (SD) of participants was 38.7 (8.6), years and the majority were female (58.3%). Participants reported an average of 15.5 (8.5) years of total nursing experience, and 9.6(8.4) years of experience in an ED setting. Most participants were staff nurses (93.2%), working on rotating shift (92.4%), and their higher education was a bachelor’s degree in nursing (62.1%). The largest proportion of participants worked in Nicosia General Hospital (22.7%). Most participants indicated that a patient triage protocol existed in their work setting (92.4%), with 59.8% stating that this was almost always implemented. However, the majority had never attended educational courses on communication skills (55.3%) or on workplace violence management (68.9%) (please see Table 1).

### 3.2. Characteristics and Context of Workplace Violence and Organizational/Administrative-Level Security Strategies for Managing Violent Incidents

There were no statistically significant associations between socio-demographic factors and violence-related variables.

#### 3.2.1. Characteristics of Workplace Violence

Approximately 92.4% of participants reported experiences of verbal violence in the last 12 months, while 16.7% reported experiences of both verbal and physical violence (please see Table 2).

Inappropriate behavior (84.1%), insult/deprecation/disrespect (62.1%) and screaming/shouting/high volume of voice (48.5%) were the most common forms of verbal violence. The most frequently reported physical violence behaviors were pushing (9.8%), pulling/grasping (6.1%), and spitting (4.5%). Armed threats with real or hand-held weapons were limited to three incidents, with one stabbing incident reported. The arm/forearm (6.1%) and hand/palm (5.3%) were the most commonly injured body parts, mainly due to scratches/abrasions (7.6%) and exposure to liquids (3.8%) (please see Table S1).

#### 3.2.2. Characteristics of Perpetrators

Patients and their escorts—both at the same time—were the most frequently reported perpetrators (32.6%), followed by patients’ escorts-solely (18.2%), and patients themselves-solely (17.4%). Long waiting times (45.5%), overcrowding in the ED (35.6%) and feelings of inattention from healthcare professionals (20.5%) were the main triggers for violent behaviors by ED service users reported by the participants. Among clinical conditions, patients’ intoxication (29.5%) and confusion/agitation/anxiety (26.5%) were the most frequently reported risks for aggressive or violent behavior by them. Most of the violence-related incidents took place during assessment and physical examination (39.4%). Consequently, the triage area (37.1%) and examination rooms (30.3%) were the places where violent episodes occurred most frequently (please see Table 3 and Table S1).

Intervention by a peer (29.5%), the security/police service (29.5%), or the participant alone trying to manage the violent behavior (27.3%) were the most common ways of managing these incidents (please see Table 3). Regarding the available architectural, regulatory, and administrative security measures available in the ED, approximately one in three participants (31.8%) reported lack of any such measures. One out of five mentioned a closed-circuit surveillance system (19.7%) and adequate lighting throughout the ED (18.2%). Overall, architectural, regulatory, and administrative safety measures were described as inadequate (please see Table 3 and Table S1).

### 3.3. Reporting Systems

Regarding systems and procedures for reporting violent incidents, approximately one-third of participants (32.6%) stated that they were unaware of the hospital’s availability of violence incident reporting procedures/systems, while one out of four (22.7%) reported a lack of violence incident reporting procedures/systems.

Reporting of violent incidents took place mostly verbally (63.6%), while only one out six proceeded to a formal written report (16.7%). Indeed, a specific violent reporting form was completed by the vast minority of the participants, while one out of six did not report at all (15.9%) (please see Table 3, Table 4 and Table S1). Regarding the person to whom the reporting took place, this was the shift supervisor (62.1%), a peer (37.9%) or the head of the ED (34.1%).

### 3.4. Self-Perceived Resilience Scores

Based on a distribution in quartiles, almost one in two participants demonstrated moderate to high mean scores (SD) of self-perceived resilience [54.25(15.0)–73.75(15.0)] and 25% of them reported a very high score of self-perceived resilience, specifically a mean value higher than 73.

#### Association Between Self-Perceived Resilience Scores and Professional Attitudes After Exposure to Workplace Violence

Participants who reported that exposure to workplace violence decreased “quite a lot to a lot” their work-related motivation presented statistically significantly lower self-perceived resilience scores [M(SD): 58.7(14.6)] compared to those who stated “very little to not at all” impact on motivation [M(SD): 66.5(13.8)] (*t*-test, *p* = 0.005). Participants who “very often to every time” considered moving from the ED to a different work setting after a workplace violent experience also reported lower self-perceived resilience scores than those who “rarely or never” considered such a move (ANOVA, mean difference = 8.37, *p* = 0.040). Similarly, participants who actually applied for a transfer to a different work setting due to experiences of workplace violence showed lower self-perceived resilience scores [M(SD): 52.1(13.4)] compared to those who did not request transfer [M(SD): 65.5(14.4)] (*t*-test, *p* < 0.001). In line with the above, self-perceived resilience scores were significantly lower among participants who “very often to every time” considered leaving the nursing profession after a workplace violence experience compared to those who “rarely to never” considered leaving the nursing profession (ANOVA, mean difference = 12.65, *p* = 0.007). No associations were found between decreased job satisfaction due to workplace violence exposure and self-perceived resilience scores (*p* = 0.154). Differences in self-perceived resilience mean scores in relation to workplace violence exposure variables are presented in Table S2.

### 3.5. Factors Related to Turnover Intention

The association between factors related to turnover intention are presented in Figure S1.

#### 3.5.1. Model 1: Considering Leaving the Nursing Profession

Participants who considered that their work motivation decreased “quite a lot to very much” after exposure to workplace violence were approximately 80 times more likely to consider leaving the nursing profession compared to those whose work motivation decreased “very little or not at all” [OR (95%CI): 79.14(17.73–353.18); *p* < 0.001]. Similarly, individuals who had not attended communication training courses/programs were about 4 times more likely to consider leaving the nursing profession after exposure to workplace violence than those who had attended such education [OR (95%CI): 4.19(1.08–16.24); *p* = 0.038].

No other variables (gender, age, province, education level, form of workplace violence experience, self-perceived resilience, workload) were statistically significantly associated with intention to leave the nursing profession (*p* > 0.05) (please see Table 5).

#### 3.5.2. Model 2: Considering Transferring to a Different Work Setting

The only predictive factor that entered the final statistical model regarding intention to move to a different work setting was decreased work motivation. Specifically, participants who reported “quite a bit to a lot” decrease in work motivation after workplace violence experience were approximately 17 times more likely to consider changing work setting compared to those who reported “very little or no” decrease [OR (95%CI): 16.97(3.78–76.15); *p* < 0.001] (please see Table 5).

#### 3.5.3. Model 3: Actual Application to Be Transferred from the ED to a Different Work Setting

A decrease in work motivation was the strongest predictor of actual turnover behavior following exposure to workplace violence.

Although self-perceived resilience was not directly linked to considering leaving the nursing profession or moving to a different work setting, it was significantly associated with actual application for a transfer to a different work setting. Specifically, each one-point increase in self-perceived resilience score resulted in a 9.4% decrease in the likelihood of requesting a transfer after exposure to workplace violence [OR (95%CI): 0.9(0.9–1.0); *p* = 0.006) (please see Table S3).

### 3.6. Open-Ended Question

Participants were invited to provide additional comments or reflections on workplace violence through an open-ended question included at the end of the questionnaire (please see Table 6). A recurring concern was the need for enhanced security measures, including continuous police presence, surveillance systems, and safer physical infrastructure within EDs. Several participants emphasized the importance of legal enforcement, including criminal prosecution and stricter visitor regulations. Others highlighted the lack of managerial support and the need for psychological assistance and structure debriefing. Additional recommendations included training programs to help nurses manage violent situation, while a few participants pointed to broader societal issues, such as limited public respect and awareness of the nursing role. Collectively, these responses reflect the multifaceted nature of workplace violence and underscore the need for coordinated, system-wide interventions.

## 4. Discussion

This study explored the multifaceted issue of workplace violence in ED nurses in the RC, with a particular focus on its associations to nurses’ self-perceived resilience, socio-demographic and vocational parameters. The present findings highlighted the pivotal role of self-perceived resilience in mitigating adverse emotional and professional consequences of workplace violence exposure, with higher resilience scores being associated with lower perceived impact on work motivation and turnover behavior, thus greater capacity to cope with violent incidents in the workplace. While these findings confirmed that resilience is associated with turnover behavior, interpreting resilient attitude solely as an individual characteristic risks oversimplifying the phenomenon. In contrast, the present findings also underscored interacting organizational factors with turnover attitudes, such as the lack of training opportunities and decreased work motivation which may constrain resilient behaviors and trigger the intention to leave the nursing profession. This suggests that resilient behaviors and turnover intention should not be understood in isolation, but rather as a dynamic process shaped mainly by institutional conditions. From this perspective, strategies to reduce turnover intention must extend beyond individual-level interventions, such as stress management or coping skills training, and address organizational responsibilities, including the establishment of supportive reporting mechanisms, training in violence prevention, and broader institutional commitments to workplace safety, to enhance work-related motivation and allow resilient behaviors to flourish.

Overall, this study makes a novel contribution by simultaneously examining the prevalence and reporting of workplace violence, its organizational context, and its relationship with resilience and turnover among emergency nurses in the RC. While previous research has primarily emphasized either the prevalence of violence [45,47], the individual coping strategies and resilience of healthcare workers [71], or institutional support [69,76,77] our findings integrated both perspectives, demonstrating how resilience interacts with institutional support, organizational factors, and turnover attitudes within the context of exposure to workplace violence. To our knowledge, this is the first post-pandemic study in the Mediterranean region to provide empirical quantitative evidence on the link between exposure to workplace violence, resilience, turnover intentions and actual turnover behaviors, thereby offering new insights into how workplace violence translates into workforce instability in the post-pandemic period. By situating resilience within its broader organizational context, the study provides advanced understanding of workplace violence as both an individual and systemic challenge, underscoring the need for multifaceted post-pandemic interventions.

### 4.1. Workplace Violence Characteristics

Regarding violent phenomena, high rates of verbal violence were indicated, consistent with global findings [12,84,85]. However, the prevalence observed was notably higher than global averages reported in meta-analyses [12] and aligned with earlier findings from Cyprus [73]. These discrepancies may partly be attributed to underreporting, a common issue in workplace violence research [12,73]. Indeed, incidents in this study were often reported verbally, primarily to peers or shift supervisors, and rarely escalated to formal documentation. This pattern may be influenced by the perceived normalization of violence in ED work environment, limited awareness of formal reporting systems, and inadequate security measures, all of which likely discourage proper reporting and complicate prevention and management efforts.

Although international literature identified socio-demographic and occupational risk factors, including age, gender, work experience, and shift patterns [12,85], no significant associations were found in this study. This might be explained by gender imbalance in the sample, limiting the statistical power for certain comparisons. Previous studies have reported that younger, less experienced healthcare workers, and those working long or night shifts, were at greater risk of being exposed to violence [84]. Discrepancies may reflect contextual and methodological differences across studies, such as sample composition and healthcare system structures.

Violence was predominantly perpetrated by patients and their companions, often influenced by clinical and environmental stressors such as intoxication, mental health disturbances, overcrowding, and long waiting times [73,84]. The triage area and examination rooms were identified as the highest-risk locations, reflecting the vulnerability of frontline spaces where patient emotions often run high. Insufficient security infrastructure exacerbated this risk, with one-third of participants reporting no security measures in place. This highlighted the need for architectural, regulatory, and administrative safeguards, such as locked doors, metal detectors, clear signage, bright lighting, and transparent workspace designs [27].

A notable finding was the high proportion of participants who had never attended training programs on communication or workplace violence management. The present open-ended responses emphasized the need for targeted educational opportunities. Structured and ongoing training in de-escalation techniques, communication skills, and violence prevention strategies has been shown to improve management of aggressive behavior [86,87], underscoring the importance of integrating such programs into professional development initiatives.

### 4.2. Workplace Violence and Self-Perceived Resilience

The present study documented a clear association between workplace violence exposure, self-perceived resilience, work motivation and turnover attitudes in ED nurses. Specifically, the majority reported moderate resilience, with a quarter scoring very high, while those with lower resilience reported greater decrease in work motivation. In fact, participants experiencing substantial decrease in motivation were nearly 80 times more likely to consider leaving the profession and more likely to seek a transfer to a different work setting. Most importantly, participants with higher self-perceived resilience were significantly less likely to request a transfer to a different work setting after experiencing workplace violence. These findings support the notion that resilience may be a protective factor against emotional exhaustion and loss of motivation following workplace adversity for emergency nurses, as previously suggested [57,85,88,89,90,91,92].

Surprisingly, self-perceived resilience was not associated with job satisfaction decrease following workplace violence exposure, suggesting that job satisfaction may depend on broader factors, such as organizational culture, management style, professional recognition and status, welfare and salary, and promotion opportunities [57,93]. While resilience helps maintain emotional balance, it may not buffer against dissatisfaction arising from systemic or structural workplace issues. Thus, efforts to strengthen resilience should be accompanied by organizational reforms to improve professional outcomes [57,94,95].

Regarding the type of violence experienced, no differences in resilience scores were observed between those exposed to verbal and/or physical violence and those unexposed, suggesting that resilience is a personal trait not directly influenced by isolated violent episodes [96]. Nevertheless, organizational support has been shown to enhance resilience independently of the degree of exposure to violence [97,98]. Indeed, the participants who reported the existence of formal reporting systems exhibited higher resilience scores than those without. This suggests that institutional structures may reinforce individual resilience, possibly by fostering security and trust [99,100,101]. Consistent with prior research, resilience is a modifiable trait that can be strengthened through targeted interventions, such as communication skills and de-escalation interventions training [49,53]. Indeed, the participants who had attended communication training were four times less likely to consider leaving the nursing profession after workplace violence exposure, emphasizing the protective role of resilience, also confirming previous data [102,103].

Overall, the present findings underscore the importance of incorporating resilience-enhancing interventions into nursing education and ongoing professional development. Programs focusing on conflict resolution, de-escalation strategies, and emotional regulation are expected to prevent escalation during violent encounters and strengthen nurses’ capacity to recover from such events [86,87]. Based on previous data, resilience seems to buffer the emotional and professional impact of violence; nurses with higher resilience reported greater emotional stability, reduced moral distress, increased job satisfaction [104,105], improved professional performance, stronger sense of purpose and work engagement [85].

### 4.3. Study Limitations and Related Implications for Future Studies

Despite the novelty of the present study, its cross-sectional design does not allow for causal inferences to be drawn between workplace violence, resilience, and turnover intention. While significant associations were identified, the temporal sequence of these relationships cannot be determined. Future longitudinal research is therefore needed to clarify potential causal mechanisms and better capture changes over time. Similarly, a limitation of the analytical approach was the use of the Forward Likelihood Ratio stepwise logistic regression method. Stepwise procedures have been criticized for potential inflation of Type I error rates, instability across samples, and reliance on statistical criteria rather than theoretical frameworks. The decision to use this method was based on the exploratory aim of identifying key predictors among multiple demographic and vocational factors, and the need to avoid overfitting. Nevertheless, results should be interpreted with caution, and future studies should seek to validate these findings using hierarchical or theory-driven modeling strategies.

Additionaly, while this study employed a census sampling approach to maximize representativeness, the response rate of 53%, although effective, may limit the generalizability of the findings in different settings. Furthermore, the voluntary nature of the sampling introduces the potential for self-selection bias. Specifically, it is possible that nurses who had personally experienced workplace violence were more motivated to respond, whereas those who had not encountered such incidents may have been less inclined to participate. Conversely, some nurses with highly distressing experiences may have chosen not to respond due to discomfort in revisiting those events. These potential differences between respondents and non-respondents limit the ability to assume that the findings fully represent the perspectives of all emergency nurses in the target population. As a result, the generalizability of the study should be interpreted with caution, particularly when extrapolating to wider emergency nursing populations or other healthcare settings. Yet, according to anecdotal data of the Cyprus Nurses and Midwifery Association, the demographic, educational and personal data of the participants are similar to those reported by the participants, herein. Future research employing strategies to improve response rates and selection bias, such as mixed-method recruitment or follow-up reminders, may help reduce the impact of nonresponse bias and strengthen external validity of the reported data.

Another limitation relates to the measurement instrument. The QuINVIP16 is a structured checklist rather than a Likert-type scale. As such, conventional psychometric indices of internal consistency (e.g., Cronbach’s alpha) are not applicable [79]. Yet, the instrument has undergone cultural adaptation herein, while it has been used in previous research in its current form. Future studies would benefit from further psychometric evaluation of the instrument, such as test–retest stability or item-level analyses, which are expected to strengthen the evidence base for their use in diverse settings. In addition, collecting retrospective self-reported data may have introduced recall and reporting bias, potentially affecting the accuracy of responses. Future studies could reduce relevant limitations by incorporating prospective data collection through longitudinal cohort studies, using, for example, diary methods for data collection, or even by validating self-reports against objective records where available, or through mixed methods study designs. An alternative method would be the shortening of recall period by limiting the time frame participants are asked to recall, e.g., “in the past week” rather than “in the past year”, aiming to reduce memory distortion. Phrasing questions in standardized, well-tested questionnairs clearly is an important way to reduce ambiguity and make it easier for participants to recall accurately. Reporting bias may also have been present if participants selectively disclosed or withheld information; to address this, future research could emphasize anonymity and confidentiality more strongly, or include cross-checks with secondary data sources, e.g., personal diaries or digital activity blogs. Finally, estimation bias could have arisen from duplicate questionnaire submissions, whether in electronic or paper format. Although participants were explicitly reminded on two occasions to complete the survey only once, future studies could strengthen this safeguard by using unique identifiers or technical restrictions to prevent multiple entries.

Nevertheless, this study highlighted the need for further research on quality and safety improvement initiatives, particularly those aimed at strengthening organizational structures and motivation in the work environment. Future longitudinal and prospective studies are needed to investigate all aspects of the phenomenon, clarify the causal mechanisms of aggressive behavior, identify protective factors, and develop effective strategies to address violence against healthcare professionals. Specifically, in addition to resilience, other psychological processes may play a critical role in shaping healthcare professionals’ well-being and professional attitudes in high-stress environments, which need to be addressed in future studies. For instance, recent research with Italian ICU nurses during the COVID-19 pandemic has shown that difficulties in emotion regulation and psychological inflexibility significantly contribute to perceived stress, with effects that may be moderated by work experience [106]. These findings suggest that resilience should be considered alongside complementary mechanisms, as they are likely to interact in complex ways to influence how nurses cope with workplace violence and other occupational stressors. Thus, future studies need to include relevant psychological processes and broader theoretical frameworks.

A further limitation is that our models included only selected demographic and vocational characteristics. Other important contextual and organizational factors, such as nurse-to-patient staffing ratios, length and scheduling of shifts, prior experiences of trauma, were not measured. These unmeasured variables may also play a role in shaping both resilience and turnover intention among emergency nurses exposed to workplace violence. Future studies should incorporate such organizational and personal factors to provide a more comprehensive understanding of the determinants of resilience and workforce sustainability in high-risk emergency settings.

Moreover, our study did not assess Nursing-Sensitive Outcomes (NSOs), such as patient safety indicators (e.g., falls, medication errors, pressure injuries), which may also be affected by nurses’ exposure to workplace violence. Our focus was restricted to outcomes experienced by nurses themselves, including turnover attitudes. Future studies should examine whether and how patient-related outcomes are influenced by nurses’ exposure to violence in the ED, as this may provide a more comprehensive understanding of the broader impact of such incidents.

## 5. Conclusions

Verbal violence is the most common form experienced by ED nurses, mainly perpetrated by patients and companions. Clinical factors, long waiting times, and inadequate security measures contribute to the problem. Underreporting and lack of formal reporting systems further complicate management. Multilevel interventions are needed to prevent, address, and support staff facing workplace violence. The study emphasizes the critical role of both personal resilience and organizational support mechanisms in mitigating the negative impacts of workplace violence among ED nurses. While resilience contributes to emotional stability and reduces professional disengagement, findings underline the complex relationship between personal resources such as resilience, organizational supportive structures and professional outcomes, suggesting that interventions aimed solely at strengthening individual resilience may not be enough to preserve job satisfaction or prevent turnover intentions unless accompanied by organizational changes and motivation enhancement.

## Figures and Tables

**Table 1:** Demographic, vocational and educational characteristics of participants (N = 132).

**A. Numeric Variables**	**Mean Value (Standard Deviation)**
**Age (years)**	38.7 (8.6)
Total work experience as a nurse (years)	15.5 (8.5)
Total work experience in an ED (years)	9.6 (8.4)
**B. Categorical variables**	**% Percentage**
**Sex**	
Male	40.9
Female	58.3
**Higher level of education**	
Bachelor’s degree in Nursing or Nursing diploma	62.1
Master’s degree	33.3
Doctoral degree or Other	4.5
**Ranking**	
Registered Nurse	93.2
Head of Department	6.8
**Work shifts**	
Round the clock shift	92.4
Morning shift	7.6
**Number of participants** **who have responded from each work hospital**	
Hospital A & B	25.7
Hospital C	22.7
Hospital D & E	28.8
Hospital F & G	22.8
**Number of patients treated daily in the ED, as reported by participants**	
Up to 50 people	37.1
Up to 100 people	25.8
Up to 150 people	26.5
More than 150 people	10.6
**Implementation of a** **patient triage protocol**	
Sometimes to almost never	12.9
Most of the time	27.3
Almost always	59.8
**Attendance of educational courses on communication**	
Regularly (every 2–3 years)	9.1
Occasionally (>4–5 years)	34.1
Never	55.3
**Attendance of educational courses for healthcare workers on workplace violence management**	
Regularly (every 2–3 years)	4.5
Occasionally (>4–5 years)	24.2
Never	68.9

ED: Emergency Department.

**Table 2:** Percentage (%) of verbal and physical violence incidents reported by participants (N = 132).

Variables	Verbal Violence (%) *	Physical Violence (%) *
**Exposure to violence**		
Yes	92.4	23.5
No	7.6	76.5
**Absence from the workplace due to an incident of violence**		
Yes	3.0	0.8
No	78.0	22.7
**Number of incidents of verbal violence**		
<20 episodes	75.0	-
≥20 episodes	9.8	-
**Number of incidents of physical violence**		
≤2	-	13.6
>2	-	3.1

* Multiple answers possible; percentages may exceed 100%.

**Table 3:** Characteristics of perpetrators, the context of incidents and organizational/administrative-level security strategies for managing violent incidents (N = 132).

Variables	Responses	% *
**Perpetrator**	Patient solely	17.4
	Patients’ escorts solely	18.2
	Both at the same time patients & their escorts	32.6
**Clinical condition of perpetrator**	Dementia/Psychiatric disorder/Agitated	60.6 †
	Head injury/Substance use/Withdrawal syndrome	62.1 †
	Other/Unspecified	23.5
**Contextual factors/triggers of violent behavior**	Long wait/Crowded ED	81.1 †
	Complaints for unattendance	33.3 †
	Nothing in particular/Other	13.7
**Resources & strategies for violence management**	Staff/Security/Police presence	88.6 †
	No support/Nothing	6.8
**Security service availability**	24/7 access	56.8
	Daytime only	19.7
**Security measures in the ED**	ED entry checks/Access restrictions/Enclosed stations	31.8
	CCTV/Alarm bells/Panic buttons/Code systems	31.8
	Lighting/Room locks/Secure exits	34.8
	TVs/Food dispensers/Music/Multilingual info	22.7
	Zero-tolerance signs/Visitor ID/pass	3.8
	No measures reported	31.8

* Multiple answers possible; percentages may exceed 100%. † Some categories include multiple combined items for brevity. CCTV: Closed-Circuit Television; ED: Emergency Department; ID: Identity Document; TV: Television.

**Table 4:** Reporting systems for workplace violence incidents described by the participants (N = 132).

Variables	Responses	% *
**Incident reporting reference person**	Peer/Shift supervisor/Head of ED/Nurse manager	139.4 *
	Physician on duty	15.9
	Security service/Police	31.1
	Risk-safety/SHSO/National violence registry/Workplace safety service	6.1
	Not mentioned	8.3
**Reporting form**	Verbal (including informal reporting)	63.6
	Written (including incident forms)	28.0
	No report	15.9
**Availability of reporting procedures**	Yes	40.9
	No	22.7
	“I don’t know” response	32.6
**Responsible for violence management person/organization**	Unclear/Unknown	55.3
	Nursing staff member	13.6
	Head of ED/Nurse manager	47.0
	H&S supervisor/State occupational H&S agency/Other	11.4

* Multiple answers possible; percentages may exceed 100%. ED: Emergency Department; H&S: Health & Safety; SHSO: State Health Services Organization.

**Table 5:** Logistic regression analyses examining factors associated with intentions to change work setting or leave the nursing profession among nurses exposed to workplace violence.

Variables	Responses	%	Odds Ratio	Two-Tailed *p*-Value	95% Confidence Interval	
					Lower End	Higher End
Model 3. Actual application to be transferred from the ED to a different work setting						
Physical violence experience	Yes	22.7	0.20	0.039	0.05	0.92
Work-related motivation decrease due to workplace violence experience	Quite a lot to very much	30.3	19.64	<0.001	4.22	91.46
Overall resilience score		97.0	0.94	0.006	0.90	0.98
Model 2. Considering transferring to a different work setting						
Work-related motivation decrease due to workplace violence experience	Quite a lot to very much	30.3	16.97	<0.001	3.78	76.15
Model 1. Considering leaving the nursing profession						
Work-related motivation decrease due to workplace violence experience	Quite a lot to very much	30.3	79.14	<0.001	17.73	353.18
Attendance of communication courses	No	55.3	4.19	0.038	1.08	16.24

**Table 6:** Content analysis of open-ended question on workplace violence towards ED nurses.

Categories	Codes	% *
Exposure to violence and workplace vulnerability	Routine exposure to violence, vulnerable working conditions, lack of public awareness	32.1
Social issues and lack of public respect	Perceptions of cultural disrespect, Normalization of verbal abuse	7.1
Training and education needs	Requests for workshops, experiential programs	10.7
Security measures and environmental design	Calls for security presence, Infrastructure improvements, cameras, exits	35.7
Legal protection and enforcement	Suggestions for criminal prosecution, stricter enforcement, control of escorts	21.4
Managerial support and mental health services	Lack of support systems, Need for psychological help Need for managerial responsibility	17.9

* Some responses were multi-thematic and coded under more than one category.

## Data Availability

The data presented in this study are available on request from the corresponding author due to ethical reasons restrictions.

## Supplementary Materials

The following supporting information can be downloaded at https://www.mdpi.com/article/10.3390/healthcare13202562/s1. Figure S1: Predictors of turnover attitudes among participants exposed to workplace violence; Table S1: Characteristics of verbal and physical violence; Table S2: Logistic regression analysis of associated factors of exposure to workplace violence; Table S3: Results of logistic regression analyses examining factors associated with intentions to change work setting or leave the nursing profession among participants exposed to workplace violence.

## Abbreviations

The following abbreviations are used in this manuscript:

ACEP	American College of Emergency Physicians
APA	American Psychological Association
CCTV	Closed-Circuit Television
CDC	Centers for Disease Control and Prevention
CD-RISC	Connor-Davidson Resilience Scale
CI	Confidence Interval
CNBC	Cyprus National Bioethics Committee
COVID-19	Coronavirus Disease 2019
ED	Emergency Department
H&S	Health & Safety
ICN	International Council of Nurses
ID	Identity Document
ILO	International Labor Office
IPRC	Identification, Placement and Review Committee
IQR	Interquartile Range
NHS	National Health Service
NIOSH	National Institute for Occupational Safety and Health Administration
OR	Odd’s Ratio
OSHA	Occupational Safety and Health Administration
PSI	Public Services International
QR	Quick Response
QuIN16VIP	The 2016 Italian National Survey on Violence towards Emergency Nurses Questionnaire
RC	Republic of Cyprus
SD	Standard Deviation
SE	South-Eastern
SHSO	State Health Services Organization
TV	Television
USA	United States of America
WHO	World Health Organization
