# Supplementary material for: Workplace Violence, Self-Perceived Resilience and Associations with Turnover Intention Among Emergency Department Nurses: A Cross-Sectional Study
Source: Healthcare (Basel). 2025 Oct 11;13(20):2562. doi: 10.3390/healthcare13202562 (PMC12562557; doi:10.3390/healthcare13202562)
Supplement: Supplementary file 1 [file healthcare-13-02562-s001.zip › healthcare-3837512-supplementary.pdf]

Figure S1. Predictors of turnover attitudes among participants exposed to workplace violence. Odds ratios (OR) and 95% confidence intervals (CI) are presented for significant predictors across three models: (1) considering leaving the nursing profession, (2) considering transfer to a different work setting, and (3) actual application for transfer from the emergency department. Decreased work motivation emerged as the strongest and most consistent predictor across all models, while lack of communication training was associated with considering leaving the profession, and higher resilience scores were associated with reduced likelihood of applying for transfer. (Style should be applied to MDPI 5.1 figure caption)

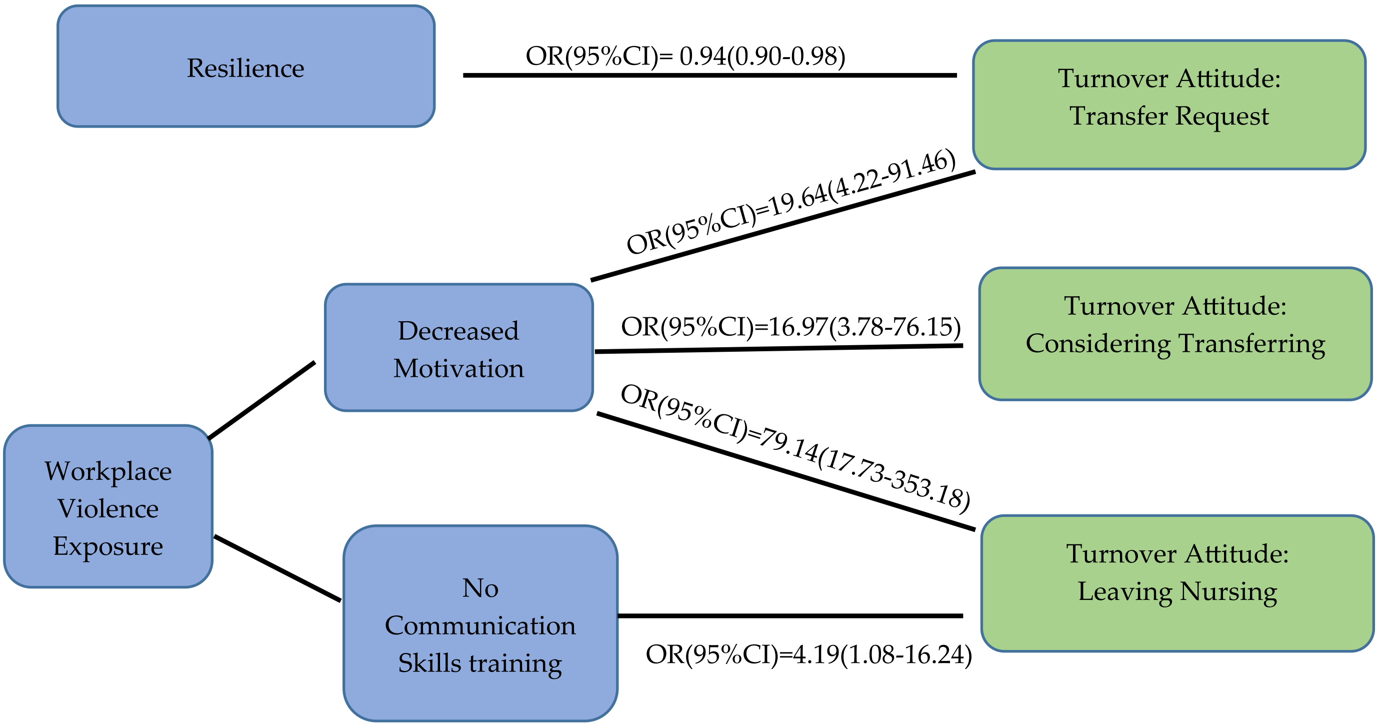

Table S1. Characteristics of verbal and physical violence (N= 132).

Variables	%*
**Exposure to verbal violence**	
Yes	92.4
No	7.6
Type of verbal violence	
Bad manners	84.1
Scurrilous language/with sexual references	8.3
Humiliation/Belittlement/Disrespect	62.1
Interruptions/Not being heeded/Being ignored	18.2
Threats of calling the police/legal action	15.9
Threats of physical aggression	22.0
Swearing and cursing	34.1
Screaming/Shouting/Being noisy	48.5
Sarcasm/Ironies	47.0
Armed threats with real weapons or weapons to hand (scissors, syringes, etc.)	2.3
Absence from the workplace due to an incident of verbal violence	
Yes	3.0

No	78.0
Number of incidents of verbal violence	
<20 episodes	75.0
≥20 episodes	9.8
**Exposure to physical violence**	
Yes	23.5
No	76.5
Type of physical violence	
Bite	2.3
Throat hold/Attempted strangulation	0.8
Pulling/Grabbing	6.1
Hair pulling	1.5
Punching or slapping	3.0
Hit by an object (e.g. chair, appliance, equipment)	0.0
Kicking	1.5
Pinching	0.0
Pushing	9.8
Scratching	0.8
Sexual abuse	0.0
Spitting	4.5
Vomitting	1.5
Stabbing	0.8
Shot	0.0
Part of injury to the body	
Head	3.0
Face	2.3
Neck	1.5
Chest	3.0
Abdomen	1.5
Arm	6.1
Hand	5.3
Shoulder	2.3
Back	0.8
Side/Buttocks	0.0
Genitals	0.0
Leg	0.8
Foot	0.8
Type of injury	
Scratches/Abrasions	7.6
Contusion/Bruising/Swelling	3.0
Sprain/Strain	0.8
Fracture	1.5

Exposure to body fluids	3.8
Internal injuries	0.0
Laceration/Cut/Wound	0.8
Other	0.8
Nothing	12.9
Absence from the workplace due to an incident of physical violence	
Yes	0.8
No	22.7
Number of incidents of physical violence	
≤2	13.6
>2	3.1
**Person who used violence**	
ED patient	17.4
Escort of the ED patient	18.2
ED patient and escort of the ED patient	32.6
**Characteristics of the person who used violence**	
Elderly person with dementia	16.7
Person with a psychiatric disorder	17.4
Anxious/Stimulant/Agitated person	26.5
Person with head injury	3.0
Drunk person	29.5
Person under the influence of drugs	16.7
Person in substance withdrawal syndrome	12.9
Person under physical restraint	0.0
Child	0.0
Other	23.5
**Conditions of violent incident**	
Patient or accompanying person after a long waiting time	45.5
Patient or accompanying person in a crowded ED	35.6
Patient or accompanying person without attendance	12.9
Patient or accompanying person complaining of lack of attention	20.5
Nothing in particular	11.4
Other	2.3
**Place where the violent incident took place**	
Hospital entrance	12.1
Hospital Exit	4.5
Hospital waiting area	20.5
Hospital reception area	10.6
Triage	37.1
Examination room	30.3
Waiting area of the ED	25.0
Observation room (short-stay hospitalization)	6.1

Isolation/Restraint room	0.8
Resuscitation/Emergency room	5.3
Stairs/Lift/Corridor	3.0
Other	1.5
**Nurse's action before the violent incident**	
Triage	34.8
Observation/Examination of the patient	39.4
Delivering bad news	0.8
Resuscitation	4.5
Carrying out an invasive procedure	8.3
Patient immobilisation/restraint	8.3
Patient transfer	3.8
Other	9.8
**Reaction of other patients or escorts**	
Indifference (no effect)	28.8
Emulation (they started to complain or become violent themselves)	11.4
Disapproval (they reproved the attacker, condemned the incident)	10.6
No one saw/heard it	7.6
**Impact on the offender**	
You managed to stop the person yourself, without any other intervention	27.3
Another nurse helped to stop the patient's violent behaviour	29.5
The security/police service managed to stop the violent behaviour of the person	29.5
The person was attended to sooner than other patients	9.1
The person left the ED immediately on their own initiative	15.9
The person was ordered to leave the ED	3.0
The person was arrested	5.3
The person was charged without being arrested	2.3
The person was admitted to the Psychiatric Unit	0.0
Other	2.3
Nothing	6.1
**Resources/Capacities to deal with an incident of violence**	
Staff member to intervene/mediate	48.5
Rapid reaction/response team	3.0
Hospital security service	40.9
Police station in hospital	43.9
Other	5.3
Nothing	6.8
**Resource availability**	
Always (24/7)	56.8
Only during the day	19.7

**Availability of architectural, regulatory security measures in the ED**	
Bulletproof/Shatterproof glass	1.5
Enclosed nursing station	5.3
Alarm bell to the hospital security service or police service	8.3
Pseudonym/Code of attack	3.0
Access restriction regulations	9.8
Examination rooms with internal locks	8.3
Examination rooms with security exit	8.3
Emergency Department entry checks (without automatic door opening)	16.7
Curved mirrors showing invisible areas	0.0
Panic button/Silent alarm	0.8
Closed surveillance system (CCTV)	19.7
Metal detector	0.0
Violence forbidden signs (Zero tolerance)	3.0
Use of pass or identification card for visitors and escorts	0.8
Bright lighting everywhere	18.2
Automatic food and beverage dispensers	6.1
Televisions or screens displaying information	9.8
Newspapers, magazines, books	0.8
Background music	4.5
Multi-language information displays	1.5
Other	7.6
Nothing	31.8
**Experience of a violent episode**	
Strong concern	43.9
Fear	28.0
Loss of self-confidence	8.3
Feeling of failure/professional incompetence	4.5
It was inevitable, it's part of the job	22.7
Distrust in the hospital administration	22.0
Distrust in administrative staff	12.1
Distrust in the director of nursing services	13.6
Vulnerable	9.8
Feeling of injustice	51.5
Anger	56.1
Feeling of shame/guilt	6.8
Discouragement/Demotivation	9.1
Justify disease-related violence (e.g. head injury, dementia, drunkenness)	17.4
**Financial costs due to a violent incident**	
Yes, to medical or legal expenses	6.1

No	81.1
**Feeling at risk or in danger**	
A lot to a great deal	60.6
Quite a bit	24.2
Not really to not at all	12.9
**Confidence in ability to manage verbal violence**	
Fairly to very confident	78.0
Not very to not at all confident	20.5
**Confidence in ability to manage physical violence**	
Fairly to very confident	42.4
Not very to not at all confident	54.5
**Reduced job satisfaction**	
A lot to a great deal	35.6
Quite a bit to not at all	51.5
**Reduction of work incentives**	
A lot to a great deal	31.1
Quite a bit to not at all	57.6
**Consider leaving the nursing profession**	
Very often to each time an incident of violence occurs	10.6
Rarely to sometimes	28.0
Never	52.3
**Consider moving to a different work setting**	
Very often to each time an incident of violence occurs	22.0
Rarely to sometimes	37.1
Never	35.6
**Apply for a transfer to a different work setting**	
Yes	13.6
No	84.8

Table S2. Mean differences in self-perceived resilience scores (with SD and 95% CI) based on workplace violence exposure, workload, availability of reporting systems, impact and turnover intention (t-test, ANOVA) (N=132).

**Workplace violence exposure variables**	**Responses**	**%**	**Mean**	**SD**	**Test**	**Two-tailed p-value**	**95% Confidence Interval**	
							**Lower end**	**Higher end**
Verbal violence experience	Yes	90.2	63.47	0.936	t-test	-9.865	10.701	0.936
	No	6.8	63.89					

Physical violence experience	Yes	22.7	60.40	13.027	t-test	0.196	-2.116	10.214
	No	74.2	64.45	15.455				
Number of verbal violence episodes experienced within the last 12 months	Less than 20 episodes	73.5	64.27	14.477	t-test	0.360	-4.842	13.212
	More than 20 episodes	9.1	60.08	18.023				
Witnessing violence towards other nurses	Verbal violence	43.9	63.98	12.625	ANOVA	0.067	60.66	67.30
	Physical violence	1.5	73.50	2.121			54.44	92.56
	Verbal and physical violence	47.0	61.65	16.618			57.42	65.87
	No	3.8	78.80	14.516			60.78	96.82
Workplace violence episode-related financial cost to participants	Yes, medical or legal expenses	6.1	64.38	14.793	t-test	0.888	-11.390	9.878
	No	79.6	63.62	14.621				
Job satisfaction decrease due to workplace violence experience	Quite a lot to a lot	34.9	61.80	15.736	t-test	0.154	-9.358	1.496
	Very little to not at all	51.5	65.74	13.333				
Work-related motivation decrease due to workplace violence experience	Quite a lot to a lot	30.3	58.70	14.620	t-test	0.005	-13.277	-2.376
	Very little to not at all	57.6	66.53	13.799				
Considered moving from the ED to a different work setting due to workplace violence experience	Very often to every time an incident of violence occurs	21.2	58.89	15.123	ANOVA	0.052	53.03	64.76
	Rarely to sometimes	37.1	63.88	13.143			60.10	67.65
	Never	34.9	67.26	14.639			62.91	71.61
Considered leaving the nursing profession due to workplace violence experience	Very often to every time an incident of violence occurs	10.6	54.14	15.316	ANOVA	0.007	45.30	62.99
	Rarely to sometimes	27.3	62.22	15.247			57.06	67.38
	Never	51.5	66.79	12.853			63.68	69.91
Actually applied for a transfer to a different work setting due to workplace violence experience	Yes	13.6	52.11	13.376	t-test	<0.001	6.257	20.604
	No	82.6	65.54	14.379				

SD: Standard Deviation

Table S3. Results of logistic regression analyses examining factors associated with intentions to change work setting or leave the nursing profession among nurses exposed to workplace violence

Consider leaving the nursing profession

		B	S.E.	Wald	df	Sig.	Exp(B)	95%CI for EXP(B)	
								Lower	Upper
Step 2[b]	Did the violent incident reduce your work motivation?(1)	4.371	.763	32.806	1	.000	79.138	17.732	353.184
	Attendance on educational communication courses(1)	1.433	.691	4.297	1	.038	4.190	1.081	16.235
	Constant	-2.611	.637	16.789	1	.000	.073		

a. Variable(s) entered on step 1: Did the violent incident reduce your work motivation?

b. Variable(s) entered on step 2: Attendance on educational communication courses.

Consider moving to a different work setting

		B	S.E.	Wald	df	Sig.	Exp(B)	95%CI for EXP(B)	
								Lower	Upper
Step 1[a]	Did the violent incident reduce your work motivation?(1)	2.832	.766	13.667	1	.000	16.971	3.782	76.152
	Constant	.059	.243	.059	1	.808	1.061		

a. Variable(s) entered on step 1: Did the violent incident reduce your work motivation?

Application for transfer to a different work setting

		B	S.E.	Wald	df	Sig.	Exp(B)	95%CI for EXP(B)	
								Lower	Upper
Step 3[c]	Have you experienced physical violence?(1)	-1.587	.768	4.277	1	.039	.204	.045	.920
	Did the violent incident reduce your work motivation?(1)	2.977	.785	14.388	1	.000	19.637	4.216	91.455
	Total resilience score	-.059	.022	7.491	1	.006	.942	.903	.983
	Constant	.639	1.340	.227	1	.633	1.895		

a. Variable(s) entered on step 1: Did the violent incident reduce your work motivation?

b. Variable(s) entered on step 2: Total resilience score.

c. Variable(s) entered on step 3: Have you experienced physical violence?
